# Increased (Pro)renin Receptor Expression in the Hypertensive Human Brain

**DOI:** 10.3389/fphys.2020.606811

**Published:** 2020-11-19

**Authors:** Minhazul Mohsin, Lucas A. C. Souza, Simindokht Aliabadi, Caleb J. Worker, Silvana G. Cooper, Sanzida Afrin, Yuki Murata, Zhenggang Xiong, Yumei Feng Earley

**Affiliations:** ^1^ Departments of Pharmacology and Physiology & Cell Biology, University of Nevada, Reno, School of Medicine, Reno, NV, United States; ^2^ Center for Molecular and Cellular Signaling in the Cardiovascular System, University of Nevada, Reno, NV, United States; ^3^ Faculty of Medicine, Tokyo Medical and Dental University, Tokyo, Japan; ^4^ Department of Pathology and Laboratory Medicine, Robert Wood Johnson Medical School, Rutgers University, New Brunswick, NJ, United States

**Keywords:** (pro)renin receptor/(P)RR, human hypertension, paraventricular nucleus of the hypothalamus, rostral ventrolateral medulla, renin-angiotensin system

## Abstract

Overactivation of the renin-angiotensin system (RAS) – a central physiological pathway involved in controlling blood pressure (BP) – leads to hypertension. It is now well-recognized that the central nervous system (CNS) has its own local RAS, and the majority of its components are known to be expressed in the brain. In physiological and pathological states, the (pro)renin receptor (PRR), a novel component of the brain RAS, plays a key role in the formation of angiotensin II (Ang II) and also mediates Ang II-independent PRR signaling. A recent study reported that neuronal PRR activation is a novel mechanism for cardiovascular and metabolic regulation in obesity and diabetes. Expression of the PRR is increased in cardiovascular regulatory nuclei in hypertensive (HTN) animal models and plays an important role in BP regulation in the CNS. To determine the clinical significance of the brain PRR in human hypertension, we investigated whether the PRR is expressed and regulated in the paraventricular nucleus of the hypothalamus (PVN) and rostral ventrolateral medulla (RVLM) – two key cardiovascular regulatory nuclei – in postmortem brain samples of normotensive (NTN) and HTN humans. Here, we report that the PRR is expressed in neurons, but not astrocytes, of the human PVN and RVLM. Notably, PRR immunoreactivity was significantly increased in both the PVN and RVLM of HTN subjects. In addition, PVN-PRR immunoreactivity was positively correlated with systolic BP (sBP) and showed a tendency toward correlation with age but not body mass index (BMI). Collectively, our data provide clinical evidence that the PRR in the PVN and RVLM may be a key molecular player in the neural regulation of BP and cardiovascular and metabolic function in humans.

## Introduction

Recent studies have reported that ~46% of United States adults of 20 years of age or older suffer from hypertension (Muntner et al., 2018). It is predicted that the global prevalence of hypertension will rise to 60% by 2025, affecting over 1.56 billion people (Kearney et al., 2005). Hypertension is one of the most important risk factors for cardiovascular diseases, which lead to stroke, myocardial infarction, congestive heart failure, and chronic kidney diseases (Nwankwo et al., 2013; Go et al., 2014). Blood pressure (BP) control mechanisms are complex and are maintained by integrated neural, humoral, and renal mechanism that collectively mediate salt retention and sympathetic activation, among other actions (Paton and Raizada, 2010; Blaustein et al., 2011; Hall et al., 2015). The circulatory renin-angiotensin system (RAS), an active focus of research since the discovery of renin more than 100 years ago, plays a pivotal role in BP control (Xu et al., 2016; Forrester et al., 2018). In addition to the circulatory RAS, various tissues throughout the body, including the brain, kidney, heart, blood vessels, lungs, adipose tissue, adrenal gland, pancreas, liver, placenta, muscle, and the eye, possess all RAS components (Nguyen et al., 2002; Paul et al., 2006; Ribeiro-Oliveira et al., 2008). Notable in this context, overactivation of the brain RAS plays a major role in neurohumoral and autonomic dysregulation, an important mechanism leading to cardiovascular and metabolic disorders, including hypertension (Basting and Lazartigues, 2017; Nakagawa et al., 2020).

The (pro)renin receptor (PRR), a novel component of the RAS encoded by the AT6AP2 gene, first cloned in 2002 (Nguyen et al., 2002), non-proteolytically activates prorenin and initiates the local production of angiotensin (Ang) II (Nguyen et al., 2002, 2004); it also mediates Ang II-independent signaling pathways (Nguyen et al., 2002; Cuadra et al., 2010; Peng et al., 2013; Xu et al., 2016). Under normal physiological conditions, the PRR is expressed at higher levels in the heart, brain, and placenta, and at comparatively lower levels in the kidney and liver (Nguyen et al., 2002). The PRR is highly expressed in mouse brain regions, including cardiovascular regulatory nuclei, such as the subfornical organ (SFO), paraventricular nucleus of the hypothalamus (PVN), supraoptic nucleus (SON), rostral ventrolateral medulla (RVLM), and nucleus tractus solitarius (NTS; Contrepas et al., 2009; Li et al., 2012; Xu et al., 2016). In the human brain, the PRR is reported to be expressed in the hypothalamus and pituitary (Takahashi et al., 2010). Recently, our research group also reported that PRR expression in the SFO is elevated in hypertensive (HTN) humans (Cooper et al., 2018).

The PVN and RVLM are two major cardiovascular regulatory nuclei that control BP. The PVN is an integrative network center that directly receives angiotensinergic and sympathoexcitatory projections from neurons in circumventricular organs in the forebrain (Dampney et al., 2018), whereas the RVLM contains sympathetic neurons that control and receive afferent inputs from the PVN. Microinjection of human (pro)renin into the PVN increases splanchnic sympathetic nerve activity in rats, an effect that is reversed by co-administration of a PRR antagonist (Huber et al., 2015). These observations suggest that (pro)renin acts through the PRR in the PVN to play a role in regulating splanchnic sympathetic nerve activity and, potentially, downstream metabolic organs. A recent study reported that neuronal PRR activation is a novel mechanism for cardiovascular and metabolic regulation in obesity and diabetes (Worker et al., 2020). Despite evidence from preclinical studies suggesting a pivotal role for the PRR in cardiovascular diseases (Shan et al., 2010; Li et al., 2012; Huber et al., 2015; Souza et al., 2019), the clinical importance of the PRR in the PVN and RVLM has yet to be defined.

In the present study, we investigated the expression and cell-specific localization of the PRR in the human PVN and RVLM and assessed its clinical significance in HTN subjects. We detected PRR immunoreactivity in neurons in both of these regions, but not in astrocytes. Importantly, PRR expression was significantly higher in the PVN and RVLM of HTN humans compared with normotensive (NTN) subjects, and PVN-PRR immunoreactivity was significantly correlated with systolic BP (sBP) and showed a tendency toward correlation with age.

## Materials and Methods

### Human Subjects

**Figure 1:**
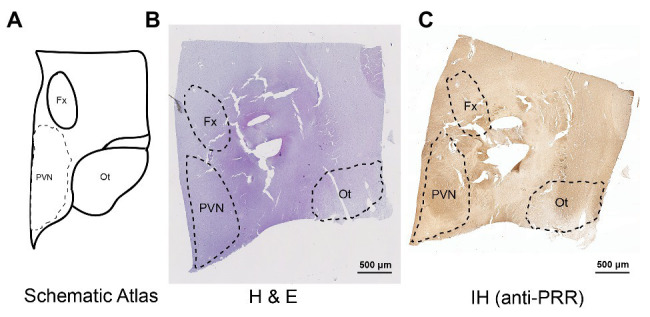
Mapping and identification of the paraventricular nucleus of the hypothalamus (PVN) in the human brain. Representative images showing the identified PVN region in a human brain section. **(A)** Schematic atlas showing the mapped location of the PVN. **(B,C)** Representative images showing hematoxylin and eosin (H&E) staining **(B)** and immunohistochemical detection of the (pro)renin receptor (PRR; **C**) in the identified PVN region of the human brain. Fx, fornics; Ot, optic tract.

**Figure 2:**
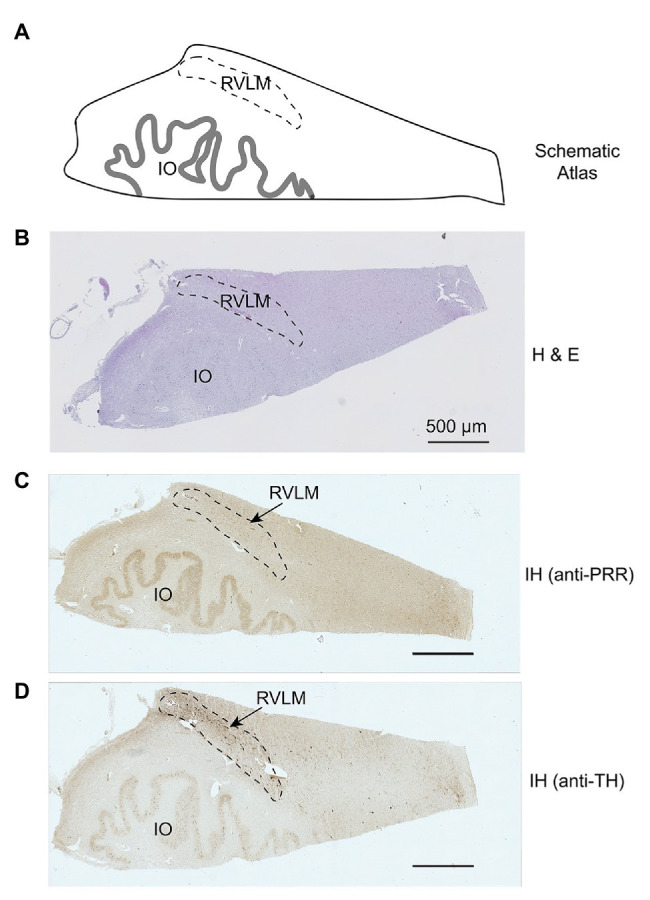
Mapping and identification of the rostral ventrolateral medulla (RVLM) in the human brain. Representative images showing the identified RVLM region in a human brain section. **(A)** Schematic atlas showing the mapped location of the PVN. **(B,C)** Representative images showing H&E staining and immunohistochemical detection of the PRR in the identified RVLM region of the human brain. **(D)** Immunohistochemical staining with an anti-tyrosine hydroxylase (TH) antibody identifying catecholaminergic neurons in the RVLM. Io, inferior olive.

A total of 23 human brains samples from autopsies were collected at the Tulane Medical Center between May 2011 and August 2015. Brain tissues were collected within 5 h of death. The PVN and RVLM brain regions were confirmed by microscopic examination using hematoxylin and eosin (H&E) staining, with reference to a human brain atlas (Ding et al., 2016). For the RVLM, we used an anti-tyrosine hydroxylase (TH) antibody to identify the C1 group of catecholaminergic neurons as an additional landmark. In the final analysis, the PVN could be identified in 11 of the 23 brain specimens (four NTNs, seven HTNs), and the RLVM could be identified in nine specimens (four NTNs, five HTNs). An example of human PVN identification is presented in Figure 1, which shows a schematic of the human PVN (Figure 1A) with H&E staining and immunohistochemical labeling of the PRR using 3,3'-diaminobenzidine (DAB) as a substrate (Figures 1B,C). Figure 2 shows a schematic illustration of the human RVLM (Figure 2A), with H&E staining and immunohistochemical labeling of the PRR and TH (Figures 2B–D). Brain tissues with no identifiable PVN or RVLM were excluded from our final analysis. All clinical data, including patient history and diagnosis, were collected, documented, and de-identified. BP used for analyses, recorded in study participants during standard care, was obtained from patient charts. The designations of hypertension and normotension used in this study are based on the individual clinical diagnosis of each human subject according to the retrospective clinical record. The next of kin consented to all autopsies and tissue collection through a standard medical autopsy protocol. This study was approved by the Institutional Review Board and the Research Integrity Offices at the University of Nevada, Reno, and complies with human research project oversight by the Institutional Review Board.

### H&E Staining

Human brain tissues containing the RVLM and PVN were dissected, fixed in 10% formalin, and embedded in paraffin. Paraffin-embedded tissues were sectioned at 5 μm thickness and mounted onto slides. Slide-mounted sections were rehydrated by two washes with xylene and two washes with 100% ethanol (10 min each) followed by washes with a graded ethanol series (95 to 80% and 80 to 70%, each for 3 min). After washing with phosphate-buffered saline (PBS; Fisher Scientific, Fair Lawn, NJ, United States) for 10 min, slides were incubated in hematoxylin for 1 min and then in eosin for 30 s; after each staining step, slides were washed in running tap water until the water ran clear. Finally, slides were dehydrated by washing with a graded ethanol series (50 to 70%, 70 to 80%, 80 to 95%, and 95 to 100%, each for 1 min).

Bright-field stitched images were captured using an optical microscope (model BZ-X710; Keyence, Elmwood Park, NJ, United States). The entire section was imaged so as to accurately identify PVN and RVLM regions by comparison with a human brain atlas (Ding et al., 2016).

### Localization of the PRR in Specific Cell Types of the Human PVN and RVLM by Immunofluorescence Labeling

Paraffin-embedded PVN and RVLM tissues were sectioned at 5 μm thickness and mounted onto slides, as described previously (Cooper et al., 2018). Briefly, the tissue was deparaffinized by heating slides in an oven at 60°C for 1 h. Sections were rehydrated by washing twice with xylene for 10 min each, followed by washing with a graded ethanol series (100 to 95% to 70 to 50 to 30%, 2 min each), and then with distilled water (dH2O) for 2 min. Antigen retrieval was performed by steaming slides in sodium citrate buffer (10 mM, pH 6.0) for 45 min. Non-specific binding was blocked by incubating with 10% goat serum for 2 h at room temperature. PVN containing sections were incubated with rabbit anti-PRR primary antibody (ab264763; Abcam, Cambridge, United Kingdom, antibody registry ID: RRID: AB_287695 at http://antibodyregistry.org), diluted 1:100 in PBS containing 0.3% Triton X-100 (PBST). PVN sections were additionally incubated overnight with mouse anti-HuC/HuD monoclonal antibody (A21271; Thermo Fisher Scientific, Waltham, MA, United States), diluted to 1:50 in PBST or chicken anti-glial fibrillary acidic protein (GFAP; ab4674; Abcam), and diluted to 1:1,000 in PBST under the same conditions. Thereafter, slides were incubated at room temperature for 2 h with Alexa Fluor 594‐ or Alexa Fluor 488-conjugated goat anti-rabbit (1:1,000), goat anti-mouse (1:1,000) or goat anti-chicken (1:1,000) secondary antibody, as appropriate. After staining with 4',6‐ diamidino-2-phenylindole (DAPI) for 5 min, all sections were incubated in TrueBlack Lipofuscin Autofluorescence Quencher (23007, Biotium Inc., Fermont, CA, United States) for 1 min to reduce the autofluorescence from aged human brain tissue. All sections were coverslip-mounted with Vectashield hard-set mounting medium (Vector Laboratories, Burlingame, CA, United States).

Rostral ventrolateral medulla sections were incubated with rabbit anti-PRR primary antibody (ab264763; Abcam) and diluted to 1:100 in PBST. RVLM sections were concomitantly incubated overnight with mouse anti-HuC/HuD monoclonal antibody (A21271; Thermo Fisher Scientific), diluted to 1:50 dilution in PBST, chicken anti-GFAP (ab4674; Abcam), and diluted to 1:1,000 in PBST. Thereafter, sections were incubated at room temperature for 2 h with Alexa Fluor 594‐ or Alexa Fluor 488-conjugated goat anti-rabbit (1:1,000), goat anti-chicken (1:1,000), or goat anti-mouse (1:1,000) secondary antibody, as appropriate. Sections were further stained with DAPI and TrueBlack following the same steps as described above for PVN sections. The specificity of all commercial antibodies used in this study was validated by negative control sections labeled without addition of primary antibodies, and data are presented in the respective immune-labeling figures. Images were captured using an epifluorescence microscope (model BZ-X710; Keyence).

### Immunohistochemical Evaluation of PRR Expression Levels in the Human PVN and RVLM

Paraventricular nucleus of the hypothalamus and RVLM tissues were fixed, paraffin-embedded, sectioned, and rehydrated, followed by antigen retrieval using Na-citrate buffer, as described above. Slides were then washed once with PBS for 5 min, placed in 3% H2O2 for 5 min, and washed in PBS for 5 min. Blockade of nonspecific binding and permeabilization was performed simultaneously by incubating with 10% goat serum in PBS containing 0.3% Triton X-100 for 1 h. For RVLM only, sections were incubated for 48 h at 4°C with the same rabbit anti-PRR antibody (diluted 1:50 in PBST containing 2% goat serum) or rabbit anti-TH polyclonal antibody (AB152; Millipore Sigma, Massachusetts, United States), diluted to 1:100 in PBST containing 2% goat serum. After a 10-min wash with PBS, sections were incubated with diluted, biotinylated rabbit IgG secondary antibody, a component of the Vectastain ABC HRP Peroxidase Kit (PK-4001; Vector Laboratories), for 30 min, followed by a 5-min wash in PBS. All slides were incubated at the same time for 2 min in a working solution of DAB Peroxidase Substrate (SK-4100; Vector Laboratories) containing 250 ml of dH2O, 4.2 ml of buffer stock solution, 4 ml of H2O2 solution, and 5 ml of DAB stock solution. The reaction was stopped by rinsing slides with tap water for 5 min, after which slides were coverslip-mounted using a xylenebased hard-set Cytoseal XYL mounting medium (Richard-Allen Scientific, Kalamazoo, MI, United States). All immunoreactions with DAB substrate, including no-primary antibody and pre-immune serum controls, were performed at the same time in the same DAB substrate developing solution.

Bright-field images were captured with an epifluorescence microscope (model BZ-X710; Keyence) using the same settings. Once the PVN or RVLM region was identified in each section, at least three to four images were captured from each section. The relative intensity of each image was analyzed using ImageJ software (version: 2.0.0-rc-68/1.52 h) and is presented as mean intensity in arbitrary units (AUs). Within each subject, the mean intensity of all images was averaged and presented as a single “*N*” value. All experiments and data analyses were performed independently in a blinded fashion by two investigators.

### Statistical Analysis

The significance of differences in sBP, diastolic BP (dBP), age, body mass index (BMI), and PRR immunoreactivity between HTN and NTN subjects was assessed with a two-tailed, unpaired Student’s *t*-test using Prism 8 software (GraphPad, La Jolla, CA, United States). Pearson correlation and least squares linear regression were used to determine correlation and regression coefficients between variables. Differences were considered statistically significant at *p* < 0.05. Multiple linear regressions were performed using SAS 9.4. All statistical tests were two-tailed, and α was set at 0.05. Data are reported as means ± SEs.

## Results

### Study Subject Characteristics

The characteristics of NTN and HTN study subjects are detailed in Tables 1, 2. All HTN subjects were clinically diagnosed as HTN with a history of taking anti-HTN medication, and all NTN subjects had a record of normal BP with no history of diagnosed hypertension. For PVN samples, study subjects in HTN and NTN groups were similar in terms of age (*p* = 0.345), body weight (*p* = 0.428), height (*p* = 0.313), and BMI (*p* = 0.899). There were similarly no significant differences in age (*p* = 0.201), body weight (*p* = 0.384), height (*p* = 0.708), or BMI (*p* = 0.560) among subjects from whom RVLM samples were obtained. HTN subjects from whom PVN samples were acquired had higher sBP (*p* = 0.001) and an increased heart rate (HR; *p* = 0.047) compared with NTN subjects. Similarly, HTN subjects from whom RVLM samples were obtained had higher sBP (*p* = 0.01) than NTN subjects; however, there was no difference in HR between groups. For both RVLM and PVN sample sets, there was no difference in dBP between HTN and NTN subjects.

**Table 1:** Characteristics of study subjects for PVN groups.

	Normotensive	Hypertensive
Region of the brain: PVN
Number of subjects/groups	4	7
Demographics:
sex, men/women	2/2	4/3
race, Caucasian/African-American	1/3	5/2
Age, years	50.5 ± 9.7	61 ± 5.8
Weight, kg	92.6 ± 8.4	104.9 ± 10.0
Height, cm	167.8 ± 3.4	177.1 ± 6.2
Body mass index, kg/m^2^	32.8 ± 2.3	33.2 ± 2.0
Last office measurements
sBP, mm/Hg	109.8 ± 8.6	144.9 ± 2.9^*^
dBP, mm/Hg	78.3 ± 10.5	78.4 ± 3.4
HR, beats/min	63 ± 21.1	113.6 ± 11.7^*^
Antihypertensive medications
Angiotensin-converting enzyme inhibitor, %	0	28.57
Diuretics, %	0	14.29
Vasodilator, %	0	42.86
No medication listed, %	0	14.29
Combination therapy, %	0	14.29

Values are means ± SEM, where appropriate; ^*^ *p* ≤ 0.05 vs. NTN by unpaired *t*-test.

**Table 2:** Characteristics of study subjects for RVLM groups.

	Normotensive	Hypertensive
Region of the brain: RVLM
Number of subjects/groups	4	5
Demographics:
sex, men/women	2/2	3/2
race, Caucasian/African-American	0/4	3/2
Age, years	51.5 ± 9.6	68.6 ± 7.6
Weight, kg	90.4 ± 10.1	111.6 ± 18.4
Height, cm	172 ± 2.8	178.2 ± 7.6
Body mass index, kg/m^2^	30.8 ± 3.9	34.8 ± 4.9
Last office measurements
sBP, mmHg	113.8 ± 4.6	146.2 ± 7.3^*^
dBP, mmHg	85.3 ± 3.6	78.6 ± 5.2
HR, beats/min	75.3 ± 24.1	112.4 ± 19.7
Antihypertensive medications
Angiotensin-converting enzyme inhibitor, %	0	20
Diuretics, %	0	20
Vasodilator, %	0	40
No medication listed, %	0	20

Values are means ± SEM, where appropriate; ^*^ *p* ≤ 0.05 vs. NTN by unpaired *t*-test.

### Localization of the PRR in Neurons of the Human PVN and RVLM

**Figure 3:**
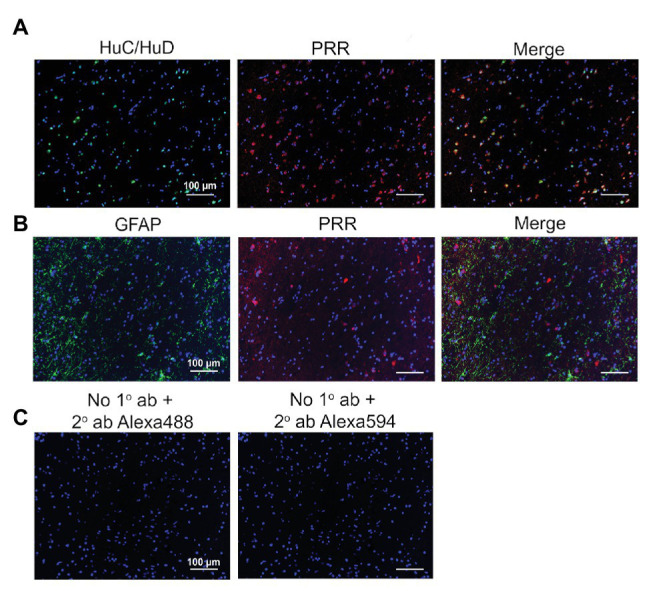
Expression of the PRR in PVN neurons of the human brain. **(A)** PVN brain tissues were immunolabeled for the PRR (red) and the neuronal marker HuC/HuD (green). **(B)** PVN brain tissues were immunolabeled for the PRR (red) and the astrocyte marker GFAP (green). **(C)** Sections processed without primary antibody but with Alexa 488 (green) or Alexa 594 (red) secondary antibody were used as negative controls. ab, antibody; 1°, primary; and 2°, secondary.

It is well-established that the PVN is a critical networking center for the angiotensinergic neural circuit in the central regulation of BP that relays excitatory pathways to the RVLM (Chen and Toney, 2003; Li et al., 2003; Cato and Toney, 2005). We recently reported that, in the human SFO, the PRR is expressed in neurons but not in astrocytes (Cooper et al., 2018). Here, we performed dual immunofluorescence labeling of the PVN for the PRR and markers of neurons or astrocytes. Within this region, we found that the PRR was colocalized with the neuronal marker HuC/HuD (Figure 3A) but not with the astrocyte marker GFAP (Figure 3B); no labeling was observed in controls (Figure 3C). These data indicate that the PRR is mostly expressed in neurons and not astrocytes in the human PVN.

**Figure 4:**
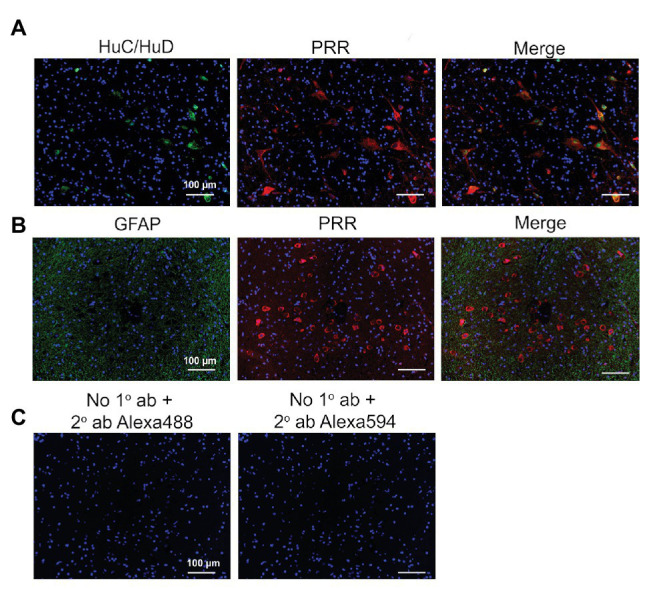
Expression of the PRR in RVLM neurons of the human brain. **(A)** RVLM brain tissues were immunolabeled for the PRR (red) and the neuronal marker HuC/HuD (green). **(B)** RVLM brain tissues were immunolabeled for the PRR (red) and the astrocyte marker glial fibrillary acidic protein (GFAP; green). **(C)** Sections processed without primary antibody but with Alexa 488 (green) or Alexa 594 (red) secondary antibody were used as negative controls. ab, antibody; 1°, primary; and 2°, secondary.

The RVLM contains sympathetic neurons that control tonic and phasic arterial pressure and receives hypothalamic afferent inputs from the PVN (Dampney, 1994; Tagawa and Dampney, 1999). Similar to the PRR expression pattern in the PVN, we found that, in the RVLM, the PRR was expressed mostly in neurons (Figure 4A) and not astrocytes (Figure 4B); again, no labeling was observed in controls (Figure 4C).

### Increased PRR Immunoreactivity in the PVN and RVLM of Hypertensive Humans

**Figure 5:**
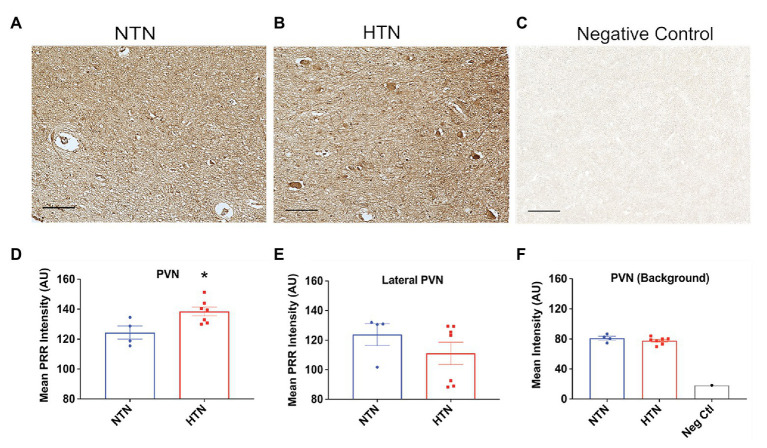
Increased PRR immunoreactivity in the PVN of the hypertensive human brain. **(A,B)** Representative images of PVN tissues from a normotensive (NTN) human brain and a hypertensive (HTN) human brain immunolabeled for the PRR. **(C)** Representative image of PVN tissue immunolabeled with rabbit pre-immune serum. **(D)** Significant difference in the relative intensity of PRR immunoreactivity in NTN and HTN groups in the PVN. **(E)** The relative intensity of PRR immunoreactivity in NTN and HTN groups in lateral PVN region. **(F)** The background relative intensity for NTN, HTN groups, and the negative control. (*N* = 7) and NTN (*N* = 4) subjects; ^*^ *p* < 0.05 vs. NTN by two-tailed, unpaired Student’s *t*-test.

**Figure 6:**
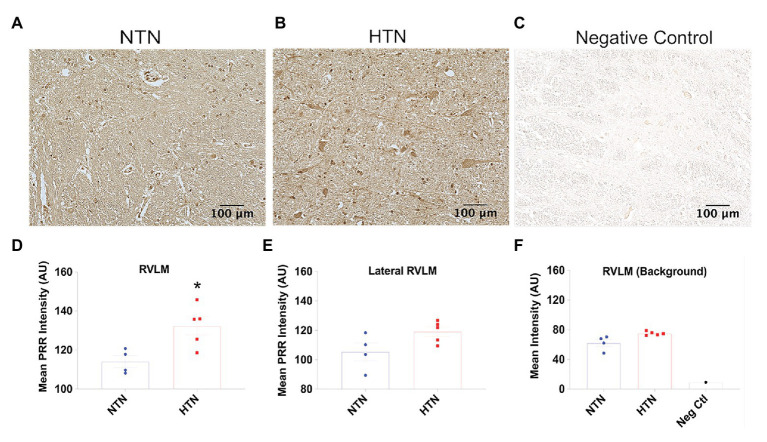
Increased PRR immunoreactivity in the RVLM of the hypertensive human brain. **(A,B)** Representative images of RVLM tissues from the NTN and HTN human brain immunolabeled for the PRR. **(C)** Representative image of RVLM tissue immunolabeled with rabbit pre-immune serum. **(D)** Significant increase in the relative intensity of PRR immunoreactivity in NTN and HTN groups in the RVLM. **(E)** The relative intensity of PRR immunoreactivity in NTN and HTN groups in lateral RVLM region. **(F)** The background relative intensity for NTN, HTN groups, and the negative control. (*N* = 5) and NTN (*N* = 4) subjects; ^*^ *p* < 0.05 vs. NTN by two-tailed, unpaired Student’s *t*-test.

With aging, human brain tissue develops a tendency to exhibit strong autofluorescence due to lipofuscin accumulation (Goyal, 1982), as we also observed in our human brain samples. Thus, to semi-quantitatively determine PRR expression levels in the PVN and RVLM, we used immunohistochemistry, presenting staining results as relative intensity in AU. Figure 5 (top panel) shows representative images of immunohistochemical labeling of the PRR in the PVN of NTN and HTN subjects, together with negative control staining without primary antibody. PRR immunoreactivity was significantly higher in HTN subjects compared with NTN subjects (*p* = 0.022), as shown in Figure 5D. To ensure the changes in PRR immunoreactivity in this brain region are specific, we also performed additional analysis of PRR immunoreactivity lateral outside the PVN and the background intensity (Figures 5E,F); we observed no difference between the NTN and HTN subjects. Figures 6A–C show representative images of PRR immunoreactivity in the RVLM of NTN and HTN subjects. We found that HTN subjects exhibited significantly higher PRR immunoreactivity compared with NTN subjects (*p* = 0.018; Figure 6D). Similarly, we analyzed the PRR immunoreactivity lateral outside the RVLM and the background intensity (Figures 6E,F); we observed no difference between the NTN and HTN subjects. The data indicate elevation in the PRR immunoreactivity in both the PVN and RVLM.

### PRR Immunoreactivity in the PVN Is Correlated With sBP in Humans

**Figure 7:**
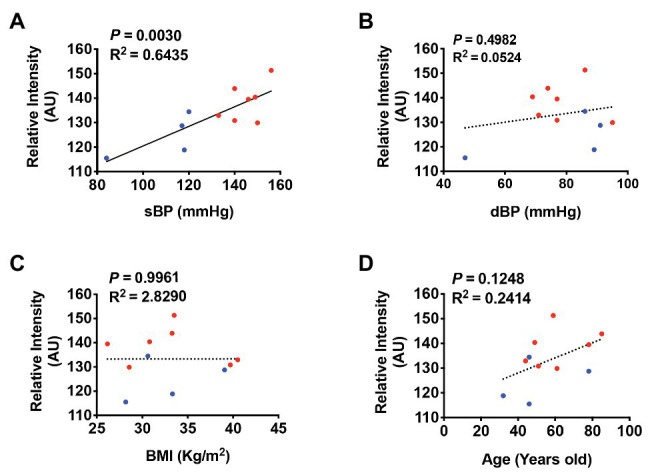
PRR immunoreactivity is positively correlated with sBP in the PVN of the human brain. A least-square correlation post analysis was used to calculate the correlation between the relative intensity of PRR immunoreactivity and systolic BP (sBP; **A**), diastolic BP (dBP; **B**), body mass index (BMI; **C**), and age **(D)**. *N* = 11.

**Figure 8:**
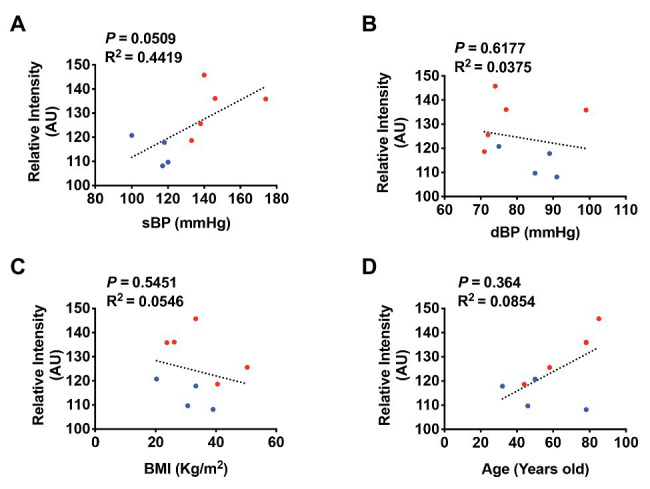
PRR immunoreactivity is not significantly correlated with sBP in the RVLM of the human brain. A least-square correlation post analysis was used to calculate the correlation between the relative intensity of PRR immunoreactivity and sBP **(A)**, dBP **(B)**, BMI **(C)**, and age **(D)**. *N* = 9.

To examine whether PRR immunoreactivity is correlated with BP, we performed a linear regression using Pearson correlation coefficient and least-square correlation post analyses. We found that PRR immunoreactivity in the PVN was significantly correlated with sBP (*R* ^2^ = 0.6435, *p* = 0.0030) but not with dBP (*R* ^2^ = 0.05243, *p* = 0.4982; Figures 7A,B). Because age, BMI, and sex are well-established factors that affect BP in humans, we also performed multiple regressions to investigate possible associations of BMI or age with PRR immunoreactivity in our study subjects. We found no significant correlation between PRR immunoreactivity in the PVN and BMI or age (Figures 7C,D). Although, there was a trend toward a positive correlation between PRR immunoreactivity and sBP in the RVLM (*R* ^2^ = 0.4416, *p* = 0.0509), this difference did not reach statistical significance (Figure 8A). There was no significant correlation between PRR immunoreactivity in the RVLM and dBP, BMI, or age (Figures 8B–D). Although not significant, the linear regression analysis showed a trend toward a positive correlation between PRR and age (Figures 7D, 8D). To further determine whether age, sex, and BMI affect the relationship between PRR and sBP, we performed a multiple regression analysis between PRR and sBP controlling for age, sex, and BMI. As showed in Table 3, PRR level in the PVN remained positively correlated with sBP (*p* = 0.015) after controlling for these parameters. In addition, there was no significant contribution of age, sex, or BMI to PRR levels in our subjects.

**Table 3:** Multiple regressions between the PRR and sBP controlling for age, BMI, and sex for PVN and RVLM groups.

Region	Variable	DF	Parameter estimate	SE	*t* value	*Pr* > |*t*|
PVN	
	Intercept	1	76.31395	20.53135	3.72	0.0099	
	Systolic blood pressure	1	0.38094	0.11297	3.37	0.015^*^	
	Body mass index	1	−0.04226	0.45232	−0.09	0.9286	
	Age	1	0.17929	0.13918	1.29	0.2451	
	Sex	1	−3.9912	4.4249	−0.9	0.4018
RVLM	
	Intercept	1	68.02497	22.59812	3.01	0.0395	
	Systolic blood pressure	1	0.36963	0.17154	2.15	0.0975	
	Body mass index	1	−0.13225	0.32799	−0.4	0.7074	
	Age	1	0.31416	0.19536	1.61	0.1831	
	Sex	1	−13.06777	6.4784	−2.02	0.1139

Parameter estimates are shown above. PVN group, *n* = 11; RVLM group, *n* = 9. ^*^ *p* < 0.05 by multiple linear regressions and Pearson correlation.

## Discussion

Sympathetic hyperactivity and autonomic dysfunction play important roles in hypertension development, cardiovascular disorder, and metabolic disorder (Mancia and Guido, 2014; Moreira et al., 2015; Xue et al., 2016). The RVLM contains a large number of sympathetic premotor neurons and receives excitatory projections from the PVN (Dampney, 1994; Tagawa and Dampney, 1999). Although previous studies reported that Ang II can access the PVN and RVLM in hypertension due to blood brain barrier breakdown (Biancardi et al., 2014; Biancardi and Stern, 2016), more recent studies have shown that the PRR represents the main source of local Ang II production in the brain (Li et al., 2014, 2015b). Accumulating evidence from experimental animal models points to a pivotal role for the brain PRR in hypertension development (Shan et al., 2010; Li et al., 2012, 2014; Souza et al., 2019). To understand the clinical significance of the brain PRR in human hypertension, we investigated PRR expression and cellular localization in the PVN and RVLM of human subjects. The key findings of this study include the following: (1) the PRR is expressed in neurons, but not astrocytes, in both the PVN and RVLM; (2) PRR immunoreactivity in both the PVN and RVLM is significantly elevated in HTN subjects; and (3) PVN-PRR immunoreactivity is positively correlated with sBP.

In rodents, the PRR is expressed in many BP-regulatory brain regions, including the SFO, SON, PVN, RVLM, and NTS (Contrepas et al., 2009; Shan et al., 2010; Li et al., 2012). In humans, PRR expression was reported in the SFO, PVN, and the pituitary (Takahashi et al., 2010; Cooper et al., 2018). PRR is colocalized with arginine vasopressin in the PVN and pituitary and with oxytocin in the hypothalamus (Takahashi et al., 2010). In the current study, we showed that the PRR is expressed in the PVN and RVLM in humans. In addition, we found that the PRR in the PVN and RVLM is predominantly expressed in neurons, and not astrocytes, a finding in accord with those of our previous study on the human SFO (Cooper et al., 2018). *In vivo* studies in rodents have similarly found that the PRR is expressed in neurons but not in astrocytes (Shan et al., 2008; Huber et al., 2015; Souza et al., 2019). This cell-type expression pattern extends to *in vitro* cell culture studies, which have also shown that the PRR is expressed in neurons but not astrocytes (Shan et al., 2010; Peng et al., 2013; Shi et al., 2014). Although these *in vitro* studies reported PRR expression in microglia, studies in mice provide no evidence for PRR localization in microglia in PVN tissue (Souza et al., 2019). On the other hand, a small number of microglia in human SFO tissue are reported to be positive for PRR immunoreactivity (Cooper et al., 2018).

An important finding from this study is that PRR immunoreactivity is significantly higher in the PVN and RVLM of HTN subjects compared with NTN subjects, indicating the potential clinical significance of the PRR in these brain regions. PVN is a part of Ang II-sensitive neural circuits (Chen and Toney, 2003; Cato and Toney, 2005; Knight et al., 2013) that receive excitatory projections from circumventricular organs in the forebrain; excitatory neurons in this brain nucleus projects to the RVLM, activating sympathetic premotor C1 neurons and increasing sympathetic activity and BP (Tagawa and Dampney, 1999; Li et al., 2003). Previous studies have demonstrated expression of the PRR in the PVN and established its role in the regulation of sympathetic activity and vasopressin secretion in NTN and HTN animals (Huber et al., 2015; Pitra et al., 2016, 2019; Souza et al., 2019). PRR expression is also upregulated in the hypothalamus of HTN mice (Li et al., 2015a). Pan-neuronal PRR knockdown or PVN-specific neuronal PRR knockdown attenuates hypertension development and high-fat diet-induced type II diabetes in mice (Souza et al., 2019; Worker et al., 2020). These studies indicate the physiological and pathological importance of the PRR in cardiovascular and metabolic regulation in rodents. However, to date, no other studies have investigated whether PRR expression is altered in the PVN of HTN humans. The current study demonstrates that PRR expression is significantly increased in the PVN of HTN subjects, suggesting the potential clinical significance of the PVN-PRR in human hypertension.

The RVLM is also known to play a significant role in the sympathetic regulation of BP, reflecting its regulation of sympathetic outflow through integration of multiple descending fibers and projections to sympathetic preganglionic neurons (Guyenet, 2006; Lohmeier and Iliescu, 2011). It has further been shown that Ang II microinjection into the mouse RVLM results in increased BP (Sasaki and Dampney, 1990), an effect that is reversed by administration of an Ang II receptor antagonist (Ito and Sved, 1996; Dampney et al., 2002; Ito et al., 2002). The PRR is present in the RVLM of mice (Contrepas et al., 2009; Li et al., 2012; Xu et al., 2016) and rats (Hu et al., 2020). In addition, blocking the PRR with the peptide PRO20 (Li et al., 2015b) attenuates neuroinflammation and decreases BP and renal sympathetic nerve activity in these animals (Hu et al., 2020), establishing a major role for the RVLM-PRR in hypertension development. Our current study demonstrates that RVLM-PRR immunoreactivity in the HTN human is significantly higher than that in NTN subjects, indicating the potential clinical importance of the PRR in the RVLM in human hypertension development. One limitation of our study is the small sample size, which initially consisted of 23 human brain specimens. However, after a careful analysis of all samples, we found that only 11 brain sections contained the defined PVN region and 9 brain sections contained defined RVLM regions. Despite the small sample size, we were able to establish significant differences between NTN and HTN groups. We would stress that the absence of a significant correlation between PRR immunoreactivity in the RVLM and sBP might be attributable to the small sample size. PVN is a heterogenous region that comprises different types of neurons. Another limitation of the current study is that we were not able to separately examine the PRR level in different type of neurons in this brain region due to technical challenges. This will be a goal of our future investigation.

In summary, we found that the PRR is prominently expressed in neurons but not astrocytes in both the PVN and RVLM. In the RVLM, the PRR is also expressed in catecholaminergic neurons. We further showed that PVN-PRR and RVLM-PRR immunoreactivity were significantly increased in HTN subjects relative to NTN subjects and that the intensity of PVN-PRR immunoreactivity was positively correlated with sBP. This study establishes the presence of the PRR in two important cardio regulatory nuclei of the human brain, namely the PVN and RVLM. The crucial role of the brain RAS in the development of hypertension is an important subject of continuing research. Expression of the PRR in neurons of the human PVN and RVLM together with previous findings in animal models of hypertension suggests the potential clinical significance of the PRR in the development of hypertension and cardiovascular and metabolic diseases in humans.

## Data Availability Statement

The raw data supporting the conclusions of this article will be made available by the authors, without undue reservation, to any qualified researcher.

## Ethics Statement

The studies involving human participants were reviewed and approved by Institutional Review Board and the Research Integrity Offices at the University of Nevada, Reno. The patients/participants provided their written informed consent to participate in this study.

## Author Contributions

YFE and MM: conceptualization. MM, CW, SC, SAl, and SAf: methodology. MM, YM, SAl, and YFE: formal analysis and investigation. MM: writing/original draft preparation. YFE, LS, and MM: writing/reviewing and editing. YFE: funding acquisition, resources, and supervision. All authors contributed to the article and approved the submitted version.

### Conflict of Interest

The authors declare that the research was conducted in the absence of any commercial or financial relationships that could be construed as a potential conflict of interest.

## References

[ref1] Basting T. Lazartigues E. (2017). DOCA-salt hypertension: an update. Curr. Hypertens. Rep. 19:32. 10.1007/s11906-017-0731-4, PMID: 28353076 PMC6402842

[ref2] Biancardi V. C. Jin S. S. Sahra A. Jessica F. A. Javier S. E. (2014). Circulating angiotensin II gains access to the hypothalamus and brain stem during hypertension via breakdown of the blood-brain barrier. Hypertension 63, 572–579. 10.1161/HYPERTENSIONAHA.113.01743, PMID: 24343120 PMC4080808

[ref3] Biancardi V. C. Stern J. E. (2016). Compromised blood-brain barrier permeability: novel mechanism by which circulating angiotensin II signals to sympathoexcitatory centres during hypertension. J. Physiol. 594, 1591–1600. 10.1113/JP271584, PMID: 26580484 PMC4799983

[ref4] Blaustein M. P. Leenen F. H. H. Chen L. Golovina V. A. Hamlyn J. M. Pallone T. L.. (2011). How NaCl raises blood pressure: a new paradigm for the pathogenesis of salt-dependent hypertension. Am. J. Physiol. Heart Circ. Physiol. 302, H1031–H1049. 10.1152/ajpheart.00899.2011, PMID: 22058154 PMC3311458

[ref5] Cato M. J. Toney G. M. (2005). Angiotensin II excites paraventricular nucleus neurons that innervate the rostral ventrolateral medulla: an in vitro patch-clamp study in brain slices. J. Neurophysiol. 93, 403–413. 10.1152/jn.01055.2003, PMID: 15356186 PMC3679885

[ref6] Chen Q. H. Toney G. M. (2003). Responses to GABA-A receptor blockade in the hypothalamic PVN are attenuated by local AT1 receptor antagonism. Am. J. Physiol. Regul. Integr. Comp. Physiol. 285, R1231–R1239. 10.1152/ajpregu.00028.2003, PMID: 12881200

[ref7] Contrepas A. Walker J. Koulakoff A. Franek K. J. Qadri F. Giaume C.. (2009). A role of the (pro)renin receptor in neuronal cell differentiation. Am. J. Physiol. Regul. Integr. Comp. Physiol. 297, R250–R257. 10.1152/ajpregu.90832.2008, PMID: 19474391 PMC2724237

[ref8] Cooper S. G. Trivedi D. P. Yamamoto R. Worker C. J. Feng C. -Y. Sorensen J. T.. (2018). Increased (pro)renin receptor expression in the subfornical organ of hypertensive humans. Am. J. Physiol. Heart Circ. Physiol. 314, H796–H804. 10.1152/ajpheart.00616.2017, PMID: 29351470 PMC5966774

[ref9] Cuadra A. E. Shan Z. Sumners C. Raizada M. K. (2010). A current view of brain renin-angiotensin system: is the (pro)renin receptor the missing link? Pharmacol. Ther. 125, 27–38. 10.1016/j.pharmthera.2009.07.007, PMID: 19723538 PMC2815255

[ref10] Dampney R. A. L. (1994). Functional organization of central pathways regulating the cardiovascular system. Physiol. Rev. 74, 323–365. 10.1152/physrev.1994.74.2.323, PMID: 8171117

[ref11] Dampney R. A. L. Fontes M. A. P. Hirooka Y. Horiuchi J. Potts P. D. Tagawa T. (2002). Role of angiotensin II receptors in the regulation of vasomotor neurons in the ventrolateral medulla. Clin. Exp. Pharmacol. Physiol. 29, 467–472. 10.1046/j.1440-1681.2002.03658.x, PMID: 12010194

[ref12] Dampney R. A. Michelini L. C. Li D.- P. Pan H. -L. (2018). Regulation of sympathetic vasomotor activity by the hypothalamic paraventricular nucleus in normotensive and hypertensive states. Am. J. Physiol. Heart Circ. Physiol. 315, H1200–H1214. 10.1152/ajpheart.00216.2018, PMID: 30095973 PMC6297824

[ref13] Ding S. -L. Royall J. J. Sunkin S. M. Ng L. Facer B. A. C. Lesnar P.. (2016). Comprehensive cellular-resolution atlas of the adult human brain. J. Comp. Neurol. 524, 3127–3481. 10.1002/cne.24080, PMID: 27418273 PMC5054943

[ref14] Forrester S. J. Booz G. W. Sigmund C. D. Coffman T. M. Kawai T. Rizzo V.. (2018). Angiotensin II signal transduction: an update on mechanisms of physiology and pathophysiology. Physiol. Rev. 98, 1627–1738. 10.1152/physrev.00038.2017, PMID: 29873596 PMC6335102

[ref15] Go A. S. Mozaffarian D. Roger V. L. Benjamin E. J. Berry J. D. Blaha M. J.. (2014). Heart disease and stroke statistics–2014 update: a report from the American Heart Association. Circulation 129, e28–e292. 10.1161/01.cir.0000441139.02102.80, PMID: 24352519 PMC5408159

[ref16] Goyal V. K. (1982). Lipofuscin pigment accumulation in human brain during aging. Exp. Gerontol. 17, 481–487. 10.1016/S0531-5565(82)80010-7, PMID: 6763901

[ref17] Guyenet P. G. (2006). The sympathetic control of blood pressure. Nat. Rev. Neurosci. 7, 335–346. 10.1038/nrn1902, PMID: 16760914

[ref18] Hall J. E. do Carmo J. M. da Silva A. A. Wang Z. Hall M. E. (2015). Obesity-induced hypertension: interaction of neurohumoral and renal mechanisms. Circ. Res. 116, 991–1006. 10.1161/CIRCRESAHA.116.305697, PMID: 25767285 PMC4363087

[ref19] Hu L. Zhang S. Ooi K. Wu X. Wu J. Cai J.. (2020). Microglia-derived NLRP3 activation mediates the pressor effect of prorenin in the rostral ventrolateral medulla of stress-induced hypertensive rats. Neurosci. Bull. 36, 475–492. 10.1007/s12264-020-00484-9, PMID: 32242284 PMC7186257

[ref20] Huber M. J. Basu R. Cecchettini C. Cuadra A. E. Chen Q. -H. Shan Z. (2015). Activation of the (pro)renin receptor in the paraventricular nucleus increases sympathetic outflow in anesthetized rats. Am. J. Physiol. Heart Circ. Physiol. 309, H880–H887. 10.1152/ajpheart.00095.2015, PMID: 26116710 PMC4591410

[ref21] Ito S. Komatsu K. Tsukamoto K. Kanmatsuse K. Sved A. F. (2002). Ventrolateral medulla AT1 receptors support blood pressure in hypertensive rats. Hypertension 40, 552–559. 10.1161/01.hyp.0000033812.99089.92, PMID: 12364362

[ref22] Ito S. Sved A. F. (1996). Blockade of angiotensin receptors in rat rostral ventrolateral medulla removes excitatory vasomotor tone. Am. J. Phys. 270, R1317–R1323. 10.1152/ajpregu.1996.270.6.R1317, PMID: 8764299

[ref23] Kearney P. M. Whelton M. Reynolds K. Muntner P. Whelton P. K. He J. (2005). Global burden of hypertension: analysis of worldwide data. Lancet 365, 217–223. 10.1016/S0140-6736(05)17741-1, PMID: 15652604

[ref24] Knight W. D. Saxena A. Shell B. Nedungadi T. P. Mifflin S. W. Cunningham J. T. (2013). Central losartan attenuates increases in arterial pressure and expression of FosB/ΔFosB along the autonomic axis associated with chronic intermittent hypoxia. Am. J. Physiol. Regul. Integr. Comp. Physiol. 305, R1051–R1058. 10.1152/ajpregu.00541.2012, PMID: 24026072 PMC3840317

[ref25] Li D. -P. Chen S. -R. Pan H. -L. (2003). Angiotensin II stimulates spinally projecting paraventricular neurons through presynaptic disinhibition. J. Neurosci. 23, 5041–5049. 10.1523/JNEUROSCI.23-12-05041.2003, PMID: 12832527 PMC6741207

[ref26] Li W. Liu J. Hammond S. L. Tjalkens R. B. Saifudeen Z. Feng Y. (2015a). Angiotensin II regulates brain (pro)renin receptor expression through activation of cAMP response element-binding protein. Am. J. Physiol. Regul. Integr. Comp. Physiol. 309, R138–R147. 10.1152/ajpregu.00319.2014, PMID: 25994957 PMC4504960

[ref27] Li W. Peng H. Cao T. Sato R. McDaniels S. J. Kobori H.. (2012). Brain-targeted (pro)renin receptor knockdown attenuates angiotensin II-dependent hypertension. Hypertension 59, 1188–1194. 10.1161/HYPERTENSIONAHA.111.190108, PMID: 22526255 PMC3375126

[ref28] Li W. Peng H. Mehaffey E. P. Kimball C. D. Grobe J. L. van Gool J. M. G.. (2014). Neuron-specific (pro)renin receptor knockout prevents the development of salt-sensitive hypertension. Hypertension 63, 316–323. 10.1161/HYPERTENSIONAHA.113.02041, PMID: 24246383 PMC3947277

[ref29] Li W. Sullivan M. N. Zhang S. Worker C. J. Xiong Z. Speth R. C.. (2015b). Intracerebroventricular infusion of the (PRO)renin receptor antagonist PRO20 attenuates deoxycorticosterone acetate-salt-induced hypertension. Hypertension 65, 352–361. 10.1161/HYPERTENSIONAHA.114.04458, PMID: 25421983 PMC4902274

[ref30] Lohmeier T. E. Iliescu R. (2011). Chronic lowering of blood pressure by carotid baroreflex activation: mechanisms and potential for hypertension therapy. Hypertension 57, 880–886. 10.1161/HYPERTENSIONAHA.108.119859, PMID: 21357283 PMC3085950

[ref31] Mancia G. Guido G. (2014). The autonomic nervous system and hypertension. Circ. Res. 114, 1804–1814. 10.1161/CIRCRESAHA.114.302524, PMID: 24855203

[ref32] Moreira M. C. D. S. Pinto I. S. D. J. Mourão A. A. Fajemiroye J. O. Colombari E. Reis Â. A. D. S.. (2015). Does the sympathetic nervous system contribute to the pathophysiology of metabolic syndrome? Front. Physiol. 6:234. 10.3389/fphys.2015.00234, PMID: 26379553 PMC4548210

[ref33] Muntner P. Carey R. M. Gidding S. Jones D. W. Taler S. J. Wright J. T.. (2018). Potential U.S. population impact of the 2017 ACC/AHA high blood pressure guideline. J. Am. Coll. Cardiol. 71, 109–118. 10.1016/j.jacc.2017.10.073, PMID: 29146532 PMC5873591

[ref34] Nakagawa P. Gomez J. Grobe J. L. Sigmund C. D. (2020). The renin-angiotensin system in the central nervous system and its role in blood pressure regulation. Curr. Hypertens. Rep. 22:7. 10.1007/s11906-019-1011-2, PMID: 31925571 PMC7101821

[ref35] Nguyen G. Burcklé C. A. Sraer J. -D. (2004). Renin/prorenin-receptor biochemistry and functional significance. Curr. Hypertens. Rep. 6, 129–132. 10.1007/s11906-004-0088-3, PMID: 15010017

[ref36] Nguyen G. Delarue F. Burcklé C. Bouzhir L. Giller T. Sraer J. -D. (2002). Pivotal role of the renin/prorenin receptor in angiotensin II production and cellular responses to renin. J. Clin. Invest. 109, 1417–1427. 10.1172/JCI14276, PMID: 12045255 PMC150992

[ref37] Nwankwo T. Yoon S. S. Burt V. Gu Q. (2013). Hypertension among adults in the United States: national health and nutrition examination survey, 2011-2012. NCHS Data Brief 133, 1–8. PMID: 24171916

[ref38] Paton J. F. R. Raizada M. K. (2010). Neurogenic hypertension. Exp. Physiol. 95, 569–571. 10.1113/expphysiol.2009.047282, PMID: 20407134

[ref39] Paul M. Mehr A. P. Kreutz R. (2006). Physiology of local renin-angiotensin systems. Physiol. Rev. 86, 747–803. 10.1152/physrev.00036.2005, PMID: 16816138

[ref40] Peng H. Li W. Seth D. M. Nair A. R. Francis J. Feng Y. (2013). (pro)renin receptor mediates both angiotensin II-dependent and -independent oxidative stress in neuronal cells. PLoS One 8:e58339. 10.1371/journal.pone.0058339, PMID: 23516464 PMC3597628

[ref41] Pitra S. Feng Y. Stern J. E. (2016). Mechanisms underlying prorenin actions on hypothalamic neurons implicated in cardiometabolic control. Mol. Metab. 5, 858–868. 10.1016/j.molmet.2016.07.010, PMID: 27688999 PMC5034613

[ref42] Pitra S. Worker C. J. Feng Y. Stern J. E. (2019). Exacerbated effects of prorenin on hypothalamic magnocellular neuronal activity and vasopressin plasma levels during salt-sensitive hypertension. Am. J. Physiol. Heart Circ. Physiol. 317, H496–H504. 10.1152/ajpheart.00063.2019, PMID: 31274353 PMC6766724

[ref43] Ribeiro-Oliveira A. Nogueira A. I. Pereira R. M. Boas W. W. V. dos Santos R. A. S. e Silva A. C. S. (2008). The renin-angiotensin system and diabetes: an update. Vasc. Health Risk Manag. 4, 787–803. PMID: 19065996 PMC2597759

[ref44] Sasaki S. Dampney R. A. (1990). Tonic cardiovascular effects of angiotensin II in the ventrolateral medulla. Hypertension 15, 274–283. 10.1161/01.HYP.15.3.274, PMID: 2303285

[ref45] Shan Z. Cuadra A. E. Sumners C. Raizada M. K. (2008). Characterization of a functional (pro)renin receptor in rat brain neurons. Exp. Physiol. 93, 701–708. 10.1113/expphysiol.2008.041988, PMID: 18326551 PMC3130537

[ref46] Shan Z. Shi P. Cuadra A. E. Dong Y. Lamont G. J. Li Q.. (2010). Involvement of the brain (pro)renin receptor in cardiovascular homeostasis. Circ. Res. 107, 934–938. 10.1161/CIRCRESAHA.110.226977, PMID: 20689062 PMC2948614

[ref47] Shi P. Grobe J. L. Desland F. A. Zhou G. Shen X. Z. Shan Z.. (2014). Direct pro-inflammatory effects of prorenin on microglia. PLoS One 9:e92937. 10.1371/journal.pone.0092937, PMID: 25302502 PMC4193744

[ref48] Souza L. A. C. Worker C. J. Li W. Trebak F. Watkins T. Gayban A. J. B.. (2019). (pro)renin receptor knockdown in the paraventricular nucleus of the hypothalamus attenuates hypertension development and AT1 receptor-mediated calcium events. Am. J. Physiol. Heart Circ. Phys. 316, H1389–H1405. 10.1152/ajpheart.00780.2018, PMID: 30925093 PMC6620680

[ref49] Tagawa T. Dampney R. A. (1999). AT1 receptors mediate excitatory inputs to rostral ventrolateral medulla pressor neurons from hypothalamus. Hypertension 34, 1301–1307. 10.1161/01.HYP.34.6.1301, PMID: 10601134

[ref50] Takahashi K. Hiraishi K. Hirose T. Kato I. Yamamoto H. Shoji I.. (2010). Expression of (pro)renin receptor in the human brain and pituitary, and co-localisation with arginine vasopressin and oxytocin in the hypothalamus. J. Neuroendocrinol. 22, 453–459. 10.1111/j.1365-2826.2010.01980.x, PMID: 20163518

[ref51] Worker C. J. Li W. Feng C. Souza L. A. C. Gayban A. J. B. Cooper S. G.. (2020). The neuronal (pro)renin receptor and astrocyte inflammation in the central regulation of blood pressure and blood glucose in mice fed a high-fat diet. Am. J. Physiol. Endocrinol. Metab. 318, E765–E778. 10.1152/ajpendo.00406.2019, PMID: 32228320 PMC7272727

[ref52] Xu Q. Jensen D. D. Peng H. Feng Y. (2016). The critical role of the central nervous system (pro)renin receptor in regulating systemic blood pressure. Pharmacol. Ther. 164, 126–134. 10.1016/j.pharmthera.2016.04.006, PMID: 27113409 PMC4942374

[ref53] Xue B. Thunhorst R. L. Yu Y. Guo F. Beltz T. G. Felder R. B.. (2016). Central renin-angiotensin system activation and inflammation induced by high fat diet sensitize angiotensin II-elicited hypertension. Hypertension 67, 163–170. 10.1161/HYPERTENSIONAHA.115.06263, PMID: 26573717 PMC4834194

